# In Vitro Repeatability and Inter-Device Agreement of Higher-Order Aberration Measurements in Scleral Lenses Using Two Hartmann–Shack Metrology Devices

**DOI:** 10.3390/s26113282

**Published:** 2026-05-22

**Authors:** Francesco Viviano, Marco Iovino, Rute J. Macedo-de-Araújo, José Manuel González-Meijome

**Affiliations:** 1 Clinical and Experimental Optometry Research Lab (CEORLab), School of Science, University of Minho, 4710-057 Braga, Portugal; rjfmaraujo@fisica.uminho.pt (R.J.M.-d.-A.); jgmeijome@fisica.uminho.pt (J.M.G.-M.); 2 Medlac S.r.l., 83100 Avellino, Italy; marco.iovino@medlac.it; 3 Physics Center of Minho and Porto Universities (CF-UM-UP), University of Minho, 4710-057 Braga, Portugal

**Keywords:** optical metrology, scleral lenses, higher-order aberrations, Hartmann–Shack wavefront sensor, Zernike polynomials, RMS

## Abstract

Scleral lenses (SLs) are increasingly incorporating complex optical designs, including front surface eccentricity (FSE) optimisation and wavefront-guided (WFG) corrections, to address residual higher-order aberrations (HOAs) in eyes with irregular corneas. Accurate in vitro optical verification of these surfaces relies on Hartmann–Shack (HS) metrology systems, yet commercially available devices differ substantially in lenslet array spatial sampling density, raising questions about their interchangeability for quality control purposes. This study evaluated the repeatability and inter-device agreement of HOA measurements in SLs obtained with two HS metrology systems with substantially different spatial sampling resolution. Sixteen SLs (four symmetric spherical, four spherical with toric periphery, four symmetric aspherical, four aspherical with toric periphery) were measured three times each using the SHSOphthalmic Cito (54 × 54 lenslet array) and SHSInspect Prio (157 × 157 lenslet array). Sphere (D) and Zernike coefficients from third to fifth radial orders were extracted for three aperture diameters (3.00, 5.00, and 7.00 mm) and analysed as root-mean-square (RMS) values by radial order and as Total HOA RMS. Both devices demonstrated excellent within-device repeatability for Sphere, RMS4, and Total HOA RMS (ICC: 0.994–1.000, CV ≤ 4%), while RMS3 and RMS5 showed moderate repeatability (ICC: 0.591–0.964, CV: 7–21%). Inter-device agreement was excellent at 5.00 and 7.00 mm (ICC: 0.950–1.000, mean bias < 0.006 μm), with a significant difference only for RMS3 at 7.00 mm aperture (*p* = 0.034). At 3.00 mm, significant systematic bias was detected for RMS4 (bias = −0.00102 μm, *p* < 0.001) and Total HOA RMS (bias = −0.00092 μm, *p* < 0.001), with the Cito underestimating values relative to the Prio. FSE design did not significantly influence inter-device differences. HS spatial sampling density influences HOA measurement accuracy in SLs at small apertures, and standardised high-resolution metrology protocols are essential to ensure accurate HOA characterisation.

## 1. Introduction

Recent technological advances in scleral lenses (SLs) have led to increasingly sophisticated geometries (freeform designs) [1,2,3] and complex optics [3,4,5,6,7,8], offering unparalleled comfort and optical clarity even in the most challenging cases [3,6,7]. Among the most clinically relevant advances in SL technology is the incorporation of wavefront-guided (WFG) optics for the correction of higher-order aberrations (HOAs) [3,4,5,6,7,8].

SLs are large-diameter rigid-gas permeable (RGP) contact lenses (CLs) designed to completely vault the cornea and limbus while resting entirely on the conjunctiva overlying the sclera [9]. SL are most commonly prescribed for visual rehabilitation in eyes with irregular corneas, with keratoconus (KC) representing the most frequent clinical indication [10]. SLs optically mask corneal irregularity through two complementary optical mechanisms: creating a smooth, regular anterior refracting surface and partially neutralising anterior corneal aberrations via refractive index matching between the post-lens fluid reservoir (*n* ≈ 1.336) and corneal tissue (*n* ≈ 1.376). Their large diameter promotes enhanced on-eye stability compared with smaller-diameter CLs [11], reducing translational decentration and rotational instability during blinking and eye movements, thereby providing a consistent and stable optical platform for successful HOA compensation [9,11]. Previous studies have reported that conventional SL neutralise approximately 60–65% of HOAs; however, residual HOAs often persist and may lead to visual symptoms such as ghosting, halos, and glare [6]. These residual HOAs are thought to arise primarily from posterior corneal optics, internal ocular structures, and lenticular aberrations, which remain unmasked by the tear reservoir [12].

Two principal strategies have been proposed to reduce residual HOAs in SLs: front surface eccentricity (FSE) optimisation and WFGSLs designs. FSE involves the introduction of controlled anterior surface eccentricity, generating progressive asphericity that partially compensates for spherical aberration induced by the irregular cornea. Clinical studies have shown that FSE values of 0.60–0.80 can reduce spherical aberration and coma in eyes with moderate-to-severe KC [13,14,15,16,17]. A retrospective analysis of 178 keratoconic eyes found that 0.60 FSE value was the most dispensed (53%), followed by 0.80 FSE (31%), demonstrating clinical preference based on visual outcomes [17]. However, FSE-based designs primarily address lower-order components of HOA structure and may be insufficient to fully compensate complex aberration patterns associated with highly irregular corneas. WFGSLs provide a fully customised optical correction strategy by targeting the unique residual aberration profile of each eye. Unlike FSE designs, which introduce a generalised aspherical modification, WFGSLs aim to correct the full residual HOA structure present in eyes with irregular corneas. Clinical evidence has reported reductions in total RMS HOAs of up to 56%, accompanied by improvement in high contrast visual acuity (HCVA) of approximately one to two lines [5,8]. The effectiveness of WFGSL designs critically depends on accurate and repeatable wavefront measurement, as well as precise manufacturing and on-eye alignment of the customised optical profile.

While both FSE and WFGSL approaches have demonstrated clinical efficacy, their successful implementation depends on micrometre-level manufacturing precision. Consequently, accurate in vitro optical verification is essential to ensure that the prescribed SLs are faithfully reproduced. First introduced in 1971 [18], Hartmann–Shack (HS) wavefront sensors have since been applied across a wide range of fields, including atmospheric turbulence compensation [19], adaptive optical microscopy of biological tissues [20], transmissive optics metrology [21], and ultrafast beam characterisation [22], as well as to the in vitro measurement of soft CLs [23] and RGP CLs [24]. HS-based optical metrology systems have therefore become essential quality control tools, offering rapid and non-invasive characterisation of CL’s optical performance. However, commercially available HS devices vary substantially in technical specifications, particularly lenslet array spatial sampling density, which may influence measurement accuracy when assessing such complex optical surfaces. Furthermore, uncertainty remains regarding the interchangeability of measurements obtained from different HS metrology platforms, especially given the likelihood that multiple systems will continue to coexist across manufacturing and research settings. Therefore, the purpose of this study was to evaluate the within-device repeatability and inter-device agreement of two HS-based optical metrology systems with substantially different spatial sampling densities in the measurement of SLs incorporating FSE designs across multiple aperture diameters. The main contributions of this work are (i) a systematic characterisation of within-device repeatability of HOA measurements across three analysis apertures for both instruments; (ii) a multi-aperture Bland–Altman analysis of inter-device agreement for HOA metrics, revealing aperture-dependent discrepancies linked to lenslet sampling density; and (iii) an assessment of whether lens design factors (FSE and peripheral toricity) influence inter-device measurement differences.

## 2. Materials and Methods

### 2.1. Instrumentation

Two optical metrology devices manufactured by Optocraft GmbH (Erlangen, Germany) were employed in this study. Both instruments utilise the HS principle [25] for wavefront sensing but differ substantially in spatial resolution. The SHSOphthalmic Cito (Cito) is equipped with a 54 × 54 lenslet array providing 2916 sampling points, while the SHSInspect Prio (Prio) features a 157 × 157 lenslet array providing 24,649 sampling points, representing an 8.45-fold difference in spatial sampling density. Technical specifications of both instruments are summarised in Table 1.

**Figure 1:**
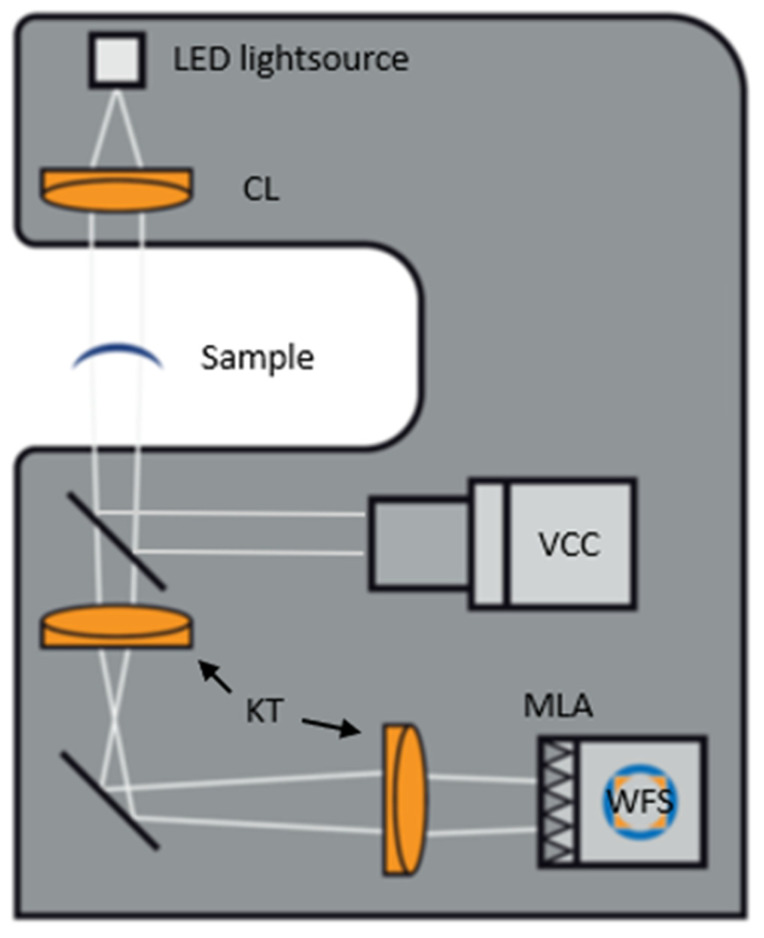
Schematic representation of the Hartmann–Shack optical metrology setup used in the SHSOphthalmic Cito and SHSInspect Prio instruments (Optocraft GmbH, Erlangen, Germany). Light emitted from the LED source passes through a collimating lens (CL) and is transmitted through the contact lens sample (Sample). A beamsplitter diverts part of the transmitted wavefront to the vision control camera (VCC), which provides a live view of the lens for centration verification, while the remaining beam is deflected by a mirror and relayed through a Keplerian telescope (KT) comprising two converging lenses, which resize and re-image the beam that continues onto the microlens array (MLA) of the wavefront sensor (WFS). The MLA samples the wavefront into multiple focal spots, enabling reconstruction of the wavefront and calculation of optical parameters, including back vertex power and Zernike aberration coefficients. LED: light-emitting diode; CL: collimating lens; KT: Keplerian telescope; MLA: microlens array; WFS: wavefront sensor; VCC: vision control camera. Adapted from Pfund, J. Contact lens measurement: beyond refractive data. GlobalCONTACT 2020, 3, 24–27.

Both instruments use identical Zernike fitting approaches: the wavefront emerging from the contact lens is sampled by the microlens array, and the displacement of focal spots on the CCD sensor is used to calculate local wavefront slopes, from which Zernike coefficients are reconstructed (Figure 1). The accompanying SHSWorks software (version: 12.002.3 (1831)) (Optocraft GmbH, Erlangen, Germany). Reconstructs the complete wavefront through numerical integration, fits it to Zernike polynomials using the OSA standard convention [26], and calculates refractive parameters, including back vertex power in air.

### 2.2. Sample Lenses

Sixteen SLs (SLC Conica, Medlac, Avellino, Italy) were custom-manufactured in Roflufocon D material (Contamac, Saffron Walden, UK). The material refractive index was 1.433 and an oxygen permeability (Dk) of 100 Fatt units. All lenses shared common specifications: total diameter (TD) of 16.80 mm, central thickness (CT) of 220 µm, and back optical zone diameter (BOZD) of 8.00 mm. The lens set was organised into four back optical zone radius (BOZR) configurations, each associated with a specific sagittal depth and nominal back vertex power, representing the spectrum typically required for fitting irregular corneas with varying degrees of steepness and elevation (Table 2).

Specifically, four design categories were evaluated: symmetric spherical (SS), symmetric aspheric with FSE = 0.70 (AS), symmetric spherical with 100 μm peripheral toricity (ST), and aspheric with FSE = 0.70 and 100 μm peripheral toricity (AT). Each design category included four lenses corresponding to the four back optical zone radius configurations (Table 2). The eccentricity value of 0.70 was based on clinical evidence from previous studies [13,14,15,16,17], all suggesting that FSE values in the range of 0.6–0.8 provide optimal visual performance and HOA reduction in irregular corneas. The peripheral toricity of 100 μm denotes a toric haptic design characterised by a sagittal height difference of 100 μm between the two principal meridians: flat (0–180°) and steep (90–270°).

### 2.3. Measurement Protocol

After manufacturing, all SLs were cleaned using Boston Laboratory Lens Cleaner (Bausch & Lomb, Rochester, NY, USA) to remove manufacturing residues and surface contaminants. Lenses were then visually inspected for manufacturing defects under an optical microscope, and central thickness was measured using a thickness gauge.

All measurements were performed by a single experienced operator in air, consistent with standard optical metrology procedures for RGP CLs. The order of lens measurements and instrument use was randomised to minimise systematic bias. For each acquisition, the lens was positioned on the instrument’s dedicated RGP lens holder, ensuring stable positioning without deformation. Lens parameters, including material refractive index, BOZR, TD, and CT, were entered into the SHSWorks (Optocraft GmbH, Erlangen, Germany) software prior to measurement. The sensor capture area was set to 8.00 mm diameter consistently across both instruments, with auto edge detection enabled. Both instruments incorporate automatic edge detection in live view mode within the SHSWorks software, which identifies the lens TD, computes its geometric centre, and displays a cross marker at that position. A second fixed cross marker identifies the centre of the sensor aperture. The operator aligned the two markers by repositioning the lens before each acquisition, ensuring centration within a tolerance of less than 0.1 mm. Three independent measurements were acquired per instrument per lens. Between consecutive acquisitions, the lens was removed from the holder, cleaned with a lint-free optical wipe, and repositioned with realignment verification, ensuring truly independent measurements and assessing the combined variability of lens positioning and instrument measurement. Half of the lenses were measured first on Cito, then on Prio, while the remaining half followed the reverse order. For each measurement, Sphere and HOAs from the 3rd to 5th radial orders were calculated. HOAs were analysed as root-mean-square (RMS) by radial order (RMS3, RMS4, RMS5) and as Total HOA RMS (3rd–5th order combined). From each 8.00 mm acquisition, Zernike coefficients were subsequently rescaled to analysis apertures of 3.00, 5.00, and 7.00 mm for comparative analysis.

### 2.4. Statistical Analysis

All data were analysed using SPSS Statistics v.27 (IBM Corp., Armonk, NY, USA) [27]. The Shapiro–Wilk test assessed the normality of continuous variables. Within-instrument repeatability was evaluated using within-subject standard deviation (Sw), coefficient of variation (CV%), repeatability coefficient (2.77 × Sw), and intraclass correlation coefficient (ICC). Inter-instrument agreement was assessed using Bland–Altman analysis (bias and 95% limits of agreement), ICC, and paired *t*-tests or Wilcoxon signed-rank tests depending on data distribution. Analyses were stratified by measurement aperture (3 mm, 5 mm, 7 mm). Two-way ANOVA examined the effects of front surface eccentricity (spherical vs. aspheric) and peripheral design (symmetric vs. toric) on inter-instrument differences at each aperture, with a Bonferroni-corrected post hoc test when significant. Statistical significance was set at α = 0.05.

## 3. Results

Within-device repeatability results are summarised in Table 3 for each device and each aperture. For Sphere, both metrology systems demonstrated excellent repeatability across all apertures, with ICC ≥ 0.9999 and Sw ranging from 0.001 to 0.008 D. The Prio showed consistently lower Sw at 3.00 mm (0.0026 D vs. 0.0080 D for the Cito), indicating improved positional stability at small apertures. For fourth-order RMS (RMS4) and Total HOA RMS, repeatability was excellent for both devices (ICC 0.994–1.000, CV% < 4% at all apertures). In contrast, third-order RMS (RMS3) showed moderate repeatability (ICC 0.808–0.942), with higher relative variability (CV% 11–18%), particularly at smaller apertures. Repeatability coefficients remained below 0.018 μm across all apertures. Fifth-order RMS (RMS5) exhibited greater variability (ICC 0.591–0.964, CV% 7–21%).

Although the longer acquisition time of the Prio (up to 1.0 s vs. <0.2 s for the Cito) could theoretically increase susceptibility to environmental micro-vibrations, repeatability data did not reveal any associated increase in variability, likely reflecting the stable laboratory conditions under which measurements were performed.

**Figure 2:**
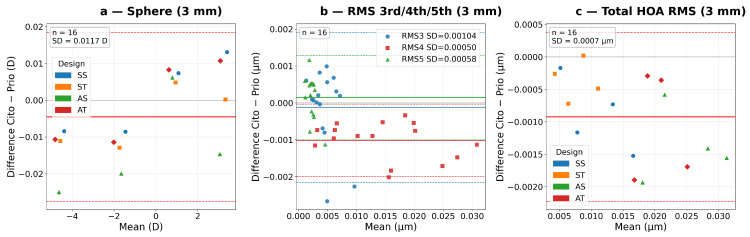
Bland–Altman plots for inter-device agreement (SHSOphthalmic Cito−SHSInspect Prio) at 3.00 mm analysis aperture. (**a**) Sphere (D); (**b**) 3rd/4th/5th HOA RMS (μm); (**c**) Total HOA RMS (μm). Solid red line: mean bias; dashed red lines: 95% limits of agreement (±1.96 SD). Point colour and shape indicate lens design (SS: spherical symmetric; ST: spherical toric; AS: aspherical symmetric; AT: aspherical toric). *n* = 16 lenses.

**Figure 3:**
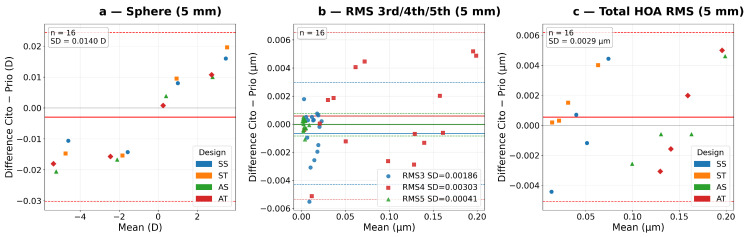
Bland–Altman plots for inter-device agreement (SHSOphthalmic Cito−SHSInspect Prio) at 5.00 mm analysis aperture. (**a**) Sphere (D); (**b**) 3rd/4th/5th HOA RMS (μm); (**c**) Total HOA RMS (μm). See Figure 2 for legend details.

**Figure 4:**
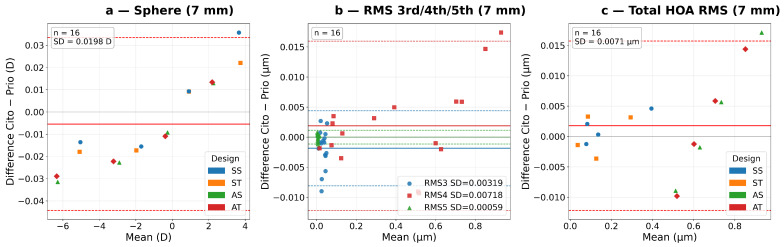
Bland–Altman plots for inter-device agreement (SHSOphthalmic Cito−SHSInspect Prio) at 7.00 mm analysis aperture. (**a**) Sphere (D); (**b**) 3rd/4th/5th HOA RMS (μm); (**c**) Total HOA RMS (μm). See Figure 2 for legend details.

Inter-device agreement results are summarised in Table 4:

Inter-device agreement results are illustrated in Figure 2, Figure 3 and Figure 4:

At 5.00 and 7.00 mm analysis apertures, agreement between instruments was generally good with ICC values ≥ 0.950 for all metrics. A statistically significant mean difference was observed only for RMS3 at 7.00 mm (bias = −0.00185 μm, *p* = 0.034, ICC = 0.964), while all other metrics showed no significant mean differences (*p* > 0.034).

In contrast, at 3.00 mm, a statistically significant systematic bias was identified for RMS4 (bias = −0.00102 μm, 95% LoA [−0.002, −0.00003], *p* < 0.001, ICC = 0.991) and Total HOA RMS (bias = −0.00092 μm, 95% LoA [−0.002, +0.000], *p* < 0.001, ICC = 0.991), with the Cito consistently underestimating values relative to the Prio at this aperture (Figure 2c). Bland–Altman plots further revealed a significant proportional bias for Sphere at all apertures (r = 0.682–0.927, all *p* < 0.04), indicating that inter-device differences in back vertex power increased with the magnitude of the measured value (Figure 2a, Figure 3a and Figure 4a).

The effect of lens design on inter-device differences was evaluated by two-way ANOVA (Table 5).

Lens design factors did not significantly influence inter-device differences. Neither front-surface eccentricity (FSE) nor peripheral toricity (PT) showed a significant effect on measurement agreement at 3.00 or 5.00 mm apertures for any analysed metric.

At 7.00 mm aperture, a borderline effect of FSE on fifth-order RMS differences was observed (*p* = 0.050), while no significant effects were detected for any other parameter. No significant interaction between FSE and peripheral toricity was found at any aperture.

## 4. Discussion

Both devices demonstrated excellent repeatability for Sphere (ICC > 0.994), RMS4, and Total HOA RMS (ICC > 0.994, CV% < 4%), confirming their suitability for routine SL quality control. Moderate repeatability for RMS3 (ICC 0.808–0.942, CV% 11.4–18.5%) and variable repeatability for RMS5 (ICC 0.591–0.964, CV% 7.3–21.0%) reflect the small absolute magnitude of these terms in the lens sample rather than instrument instability. The high CV% observed for RMS5 reflects a mathematical artefact of computing relative variability when absolute magnitudes approach zero, rather than true instrument instability; the repeatability coefficient for RMS5 remained below 0.003 μm across all apertures. The Prio showed lower Sw for Sphere at 3.00 mm (0.0026 vs. 0.0080 D), which may be attributable to its finer lenslet pitch improving centroid localisation during repeated positioning, though no consistent repeatability advantage was observed for RMS4 or Total HOA RMS at any aperture.

Taken together, these findings indicate that within-device repeatability is primarily influenced by aberration magnitude and analysis aperture rather than intrinsic instrument instability, with both instruments showing improved repeatability at larger apertures for higher-order RMS metrics. Inter-device agreement was excellent at 5.00 mm and 7.00 mm (ICC > 0.950, mean bias < 0.006 μm for all metrics), supporting practical interchangeability at these apertures. At 3.00 mm, a statistically significant systematic bias was identified for RMS4 and Total HOA RMS, with the Cito consistently underestimating values relative to the Prio. This directional bias is consistent with Cito’s lower spatial sampling density (54 × 54 vs. 157 × 157 lenslets), which captures fewer independent wavefront points within a 3.00 mm aperture, limiting reconstruction of higher spatial frequency Zernike components. The reduced agreement observed at smaller apertures may reflect the limited capability of lower-density HS sampling grids to resolve higher spatial frequency wavefront components, particularly when the effective number of lenslets contributing to the reconstruction becomes restricted. A significant proportional bias was additionally identified for Sphere at all apertures (r = 0.682–0.927, *p* < 0.04), indicating that inter-device differences in back vertex power scale with measurement magnitude. The proportional bias observed for spherical power across all apertures is consistent with residual systematic offsets attributable to differences in the internal optical design between the two instruments, compounded by the fact that the manufacturer’s Refractive Data Correction (RDC) calibration applies to a 4.50 mm aperture only; measurements of spherical power at 3.00, 5.00, and 7.00 mm were therefore obtained outside the designated calibration range. Notably, this limitation applies symmetrically to both instruments and does not compromise the within-device repeatability findings. Neither FSE nor TP significantly influenced inter-device differences, indicating that discrepancies are instrument-dependent rather than geometry-dependent. A preliminary analysis by our group using the same lenses and measurement protocol performed at a single aperture of 8.00 mm was recently presented and concluded full interchangeability of the two devices [28].

The overall similarity of outcomes between the two devices was to some extent anticipated, given that the SLs evaluated in this study present smooth, rotationally symmetric, or mildly aspherical optical surfaces. On such surfaces, the wavefront reconstructed from a coarser lenslet array (54 × 54, Cito) and that from a denser array (157 × 157, Prio) are expected to converge, as the spatial frequency content of the wavefront is low and well within the sampling capacity of both sensors. In contrast, it would be expected that the higher lenslet density of the Prio would provide a more accurate wavefront reconstruction for optical surfaces with rapidly varying power profiles or discrete power transitions, such as bifocal or multifocal contact lenses with alternating power ring designs, where the coarser sampling of the Cito may undersample steep local wavefront gradients. Mean RMS4 increased 28-fold from 3.00 mm to 7.00 mm (0.015 to 0.417 μm), highlighting the critical aperture dependence of HOA measurements. The present stratified analysis reveals that this conclusion holds at 5.00 and 7.00 mm but not at 3.00 mm, where the systematic bias was masked by the dominant contribution of large-aperture aberrations to total RMS. This underscores the necessity of reporting measurement aperture as a mandatory parameter in contact lens metrology. The present findings are consistent with those reported by Domínguez-Vicent et al. [29], who characterised the power profiles and optical quality of SLs across five nominal powers using the NIMO TR-1504 optical system at 3.00 and 6.00 mm apertures, reporting Total HOA RMS values below 0.05 μm at 3.00 mm and below 0.08 μm at 6.00 mm regardless of lens power or design, confirming the optically smooth nature of scleral lens surfaces and the inherently low aberration content at smaller apertures. Despite statistically significant differences in RMS among lens powers, near-diffraction-limited MTFs and visual Strehl ratios approaching unity at 3.00 mm, closely mirroring the present findings, where inter-device differences at 3.00 mm were statistically significant but clinically negligible. Importantly, differences became more apparent at 6.00 mm, where MTFs distanced from the diffraction-limited curve and visual Strehl ratios declined, reinforcing the interpretation that metrological discrepancies are more detectable at larger apertures where aberration magnitudes are inherently greater. Regarding power accuracy, deviations from nominal power of up to 0.50 D were reported, consistent with the significant sphere bias observed in the present study and suggesting that systematic power offsets are a characteristic of SL manufacturing rather than a device-specific measurement artefact.

The inter-device discrepancies observed at 3.00 mm, while statistically significant, should be interpreted in the context of the inherently low HOA magnitudes at small apertures. Salmon and van de Pol [30] reported a mean Total HOA RMS of 0.045 μm at 3.00 mm in normal healthy adult eyes, rising to 0.100 μm at 4.00 mm and 0.327 μm at 6.00 mm, demonstrating the strong non-linear dependence of HOA magnitude on aperture. Van den Berg et al. [31,32] further demonstrated that even in highly aberrated corneas such as keratoconus and post-radial keratotomy, restricting aperture to 1.6 mm reduces HOAs to levels approaching those of normal eyes. Inter-device differences in the order of 0.001 μm at 3.00 mm, as observed in the present study, are therefore unlikely to carry clinical relevance.

Lens design characteristics, specifically FSE and peripheral toric geometry, did not significantly influence inter-device agreement across most aperture-metric combinations, which is an expected finding given that peripheral geometric features are not designed to alter the optical zone aberration profile. The borderline significant effect of FSE on RMS5 at 7.00 mm (*p* = 0.050) was unexpected, as FSE primarily targets spherical aberration (Z4^0^) rather than fifth-order terms. At 7.00 mm, the analysis aperture approaches the boundary of the optical zone, where the transition between the central aspherical surface and the peripheral geometry may introduce local wavefront irregularities detectable by the higher-resolution sensor, artifactually amplifying fifth-order terms in a design-dependent manner. This result warrants further investigation with a larger sample size and a wider range of front surface eccentricities.

Several limitations of this study should be acknowledged. The lens sample comprised 16 lenses from a single manufacturer representing four design categories, which, while methodologically controlled, limits the generalisability of the findings to other lens materials with varying refractive indices, geometries, or manufacturers.

All measurements were performed in air by a single experienced operator, consistent with standard metrology protocols but not representative of the in vivo measurement environment, where tear film dynamics, on-eye fitting, and lens flexure introduce additional sources of variability. Additionally, the use of a single operator controls for inter-operator variability but limits the generalisability of the repeatability findings to multi-operator laboratory settings.

Post hoc power analysis indicated adequate power to detect the near-zero FSE effects observed at 5.00 and 7.00 mm; however, power was limited (41%) for Total HOA RMS at 3.00 mm, where a moderate effect size was observed, and a larger sample would be required to draw definitive conclusions at this aperture.

Inter-individual variability in the HOA content of the 16 lenses reflects inherent manufacturing tolerances; however, as both instruments measured the same physical lenses, this variability does not confound the inter-device agreement analysis.

Future studies should investigate whether the inter-device discrepancies observed at 3.00 mm persist across a broader range of HOA magnitudes, including WFGSLs with intentionally large HOA amplitudes, and whether the bias is consistent across different lens materials. Extension of this methodology to in vivo HS aberrometry represents a logical next step for validating the clinical translation of in vitro metrology findings. Furthermore, a direct comparison between in vitro and in vivo HOA measurements in scleral lenses, accounting for the optical effects of the post-lens fluid reservoir, internal ocular aberrations, and on-eye lens positioning, represents a natural direction for future research in this field. 

These findings have direct implications for the manufacturing and validation of WFGSLs, where accurate reproduction of complex high-spatial-frequency optical features is essential to achieve the intended visual correction.

## 5. Conclusions

Both metrology systems demonstrated excellent within-device repeatability for Sphere, RMS4, and Total HOA RMS across all apertures (ICC: 0.994–1.000, CV ≤ 4%), supporting their use in routine SL quality control. Moderate repeatability was observed for RMS3 (ICC: 0.808–0.942) and variable repeatability for RMS5 (ICC: 0.591–0.964), attributable to the small absolute magnitudes of these terms rather than instrument instability. Inter-device agreement was excellent at 5.00 and 7.00 mm (ICC: 0.950–1.000, mean bias < 0.006 μm), supporting practical interchangeability of the two instruments at clinically relevant apertures. At 3.00 mm, a small but statistically significant systematic bias was observed for RMS4 (bias = −0.00102 μm) and Total HOA RMS (bias = −0.00092 μm), with the lower-resolution Cito device (54 × 54; 2916 sampling points) consistently underestimating aberration magnitude relative to the higher-resolution Prio device (157 × 157; 24,649 sampling points) though the absolute magnitude of these differences is unlikely to carry clinical relevance. A significant proportional bias was additionally identified for Sphere across all apertures. Lens design characteristics, including FSE and peripheral toricity, did not significantly influence inter-device agreement. These findings underscore the critical influence of analysis aperture and spatial sampling density in HOA metrology of SLs and highlight the need for standardised protocols with explicit reporting of measurement conditions when comparing optical performance across different instruments.

## Figures and Tables

**Table 1:** Technical specifications of the SHSOphthalmic Cito and SHSInspect Prio HS wavefront aberrometers (Optocraft GmbH, Erlangen, Germany) used in this study.

Parameter	SHSOphthalmic Cito	SHSInspect Prio
Image	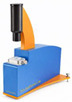	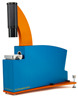
Technology	HS wavefront sensor	HS wavefront sensor
Lenslet array (lateral resolution)	54 × 54 measurement points	157 × 157 measurement points
Field of view (refractive data)	8.00 mm	8.50 mm
Wavelength	540 ± 10 nm *	540 ± 10 nm *
Spherical power range (air)	−30 to +30 D	−35 to +35 D
Power reproducibility	<0.02 D (1 σ, lens moved)	<0.02 D (1 σ, lens moved)
Power repeatability	<0.002 D (1 σ, lens not moved)	<0.002 D (1 σ, lens not moved)
Measurement duration	<0.2 s	0.2 to 1.0 s

* The 540 nm wavelength corresponds to the ISO-defined Hg-e spectral line (546.07 nm) used as the nominal wavefront measurement wavelength.

**Table 2:** Specification of scleral lenses utilised in the study and cross-sectional thickness profiles.

BOZR (mm)	Sagittal Depth (µm)	Power (D)	Thickness Profile *
9.00	4400	+3.00	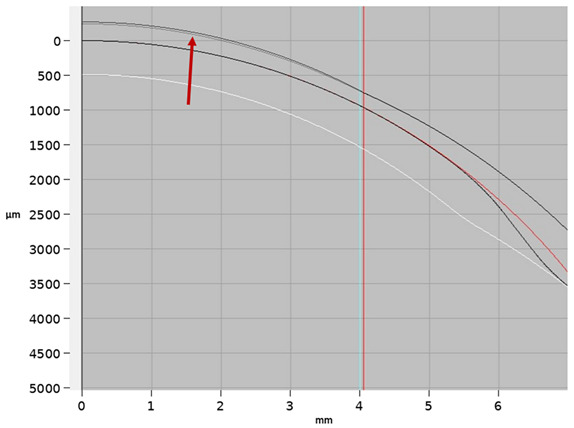
8.50	4600	+0.75	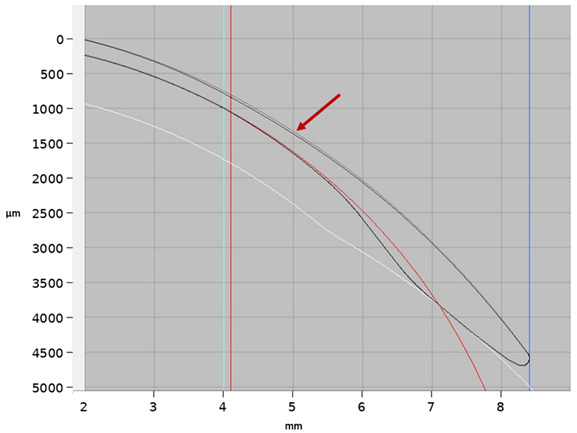
8.00	4800	−1.75	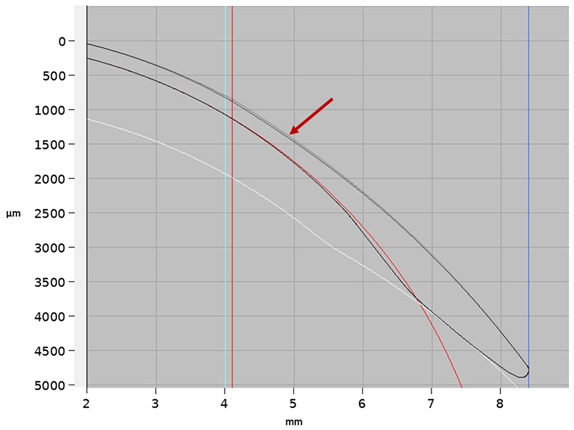
7.50	5000	−4.50	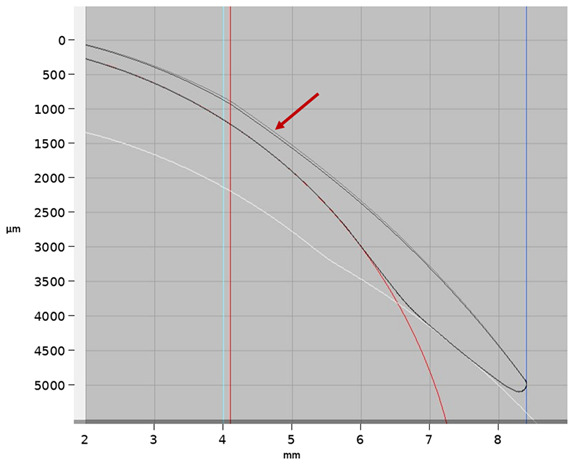

* For each BOZR configuration, the white profile represents the anterior corneal surface, the inner black profile represents the posterior lens surface, and the outer black profile represents the anterior surface of the spherical SL design. The grey profile, visible just beyond the outer black surface, represents the anterior surface of the aspherical (FSE = 0.70) SL design. The red vertical line indicates the edge of the optical zone. The red arrow highlights the region where the spherical and aspherical anterior surface profiles begin to diverge.

**Table 3:** Repeatability of SHSOphthalmic Cito and SHSInspect Prio across three measurement apertures and across all 16 lenses, encompassing four design configurations (spherical symmetric, SS; spherical toric, ST; aspherical symmetric, AS; aspherical toric, AT), with four lenses per design.

		SHSOphthalmic Cito (54 × 54)				SHSInspect Prio (157 × 157)			
Metric	Aperture (mm)	SW	CV (%)	RC	ICC	SW	CV (%)	RC	ICC
Sphere (D)	3	0.0080	—	0.0222	1.000	0.0026	—	0.0073	1.000
	5	0.0018	—	0.0051	1.000	0.0009	—	0.0024	1.000
	7	0.0022	—	0.0060	1.000	0.0015	—	0.0042	1.000
RMS 3rd order (µm)	3	0.0008	17.8	0.0022	0.842	0.0009	18.5	0.0024	0.876
	5	0.0015	11.4	0.0041	0.942	0.0019	13.8	0.0053	0.908
	7	0.0046	14.4	0.0128	0.890	0.0062	18.3	0.0173	0.808
RMS 4th order (µm)	3	0.0005	3.7	0.0015	0.996	0.0004	2.6	0.0011	0.998
	5	0.0004	0.4	0.0011	1.000	0.0021	2.3	0.0059	0.999
	7	0.0021	0.5	0.0057	1.000	0.0005	0.1	0.0014	1.000
RMS 5th order (µm)	3	0.0005	21.0	0.0014	0.591	0.0004	18.4	0.0011	0.843
	5	0.0005	15.0	0.0015	0.916	0.0005	12.7	0.0013	0.942
	7	0.0005	7.3	0.0014	0.964	0.0008	11.7	0.0022	0.909
Total HOA RMS (µm)	3	0.0006	4.0	0.0017	0.994	0.0005	3.0	0.0013	0.997
	5	0.0004	0.4	0.0011	1.000	0.0021	2.2	0.0058	0.999
	7	0.0020	0.5	0.0055	1.000	0.0007	0.2	0.0021	1.000

Sw = within-subject standard deviation; CV = coefficient of variation (not reported for Sphere as the mean power across lenses approaches zero, rendering the coefficient of variation mathematically undefined and clinically uninformative); RC = repeatability coefficient (2.77 × Sw); ICC = intraclass correlation coefficient.

**Table 4:** Inter-device agreement between SHSOphthalmic Cito and SHSInspect Prio (Bland–Altman analysis).

Metric	Aperture (mm)	Bias	LoA Lower	LoA Upper	ICC	*p*-Value
Sphere (D)	3	−0.00452	−0.02749	+0.01846	1.000	0.1442
	5	−0.00297	−0.03034	+0.02440	1.000	0.3484
	7	−0.00545	−0.04431	+0.03341	1.000	0.2889
RMS 3rd order (µm)	3	−0.00012	−0.00215	+0.00191	0.886	0.5619
	5	−0.00066	−0.00430	+0.00298	0.950	0.5619
	7	−0.00185	−0.00810	+0.00439	0.964	0.0342 *
RMS 4th order (µm)	3	−0.00102	−0.00200	−0.00003	0.991	<0.001 ***
	5	+0.00058	−0.00537	+0.00653	0.999	0.4564
	7	+0.00188	−0.01220	+0.01597	1.000	0.3108
RMS 5th order (µm)	3	+0.00015	−0.00099	+0.00129	0.756	0.3258
	5	−0.00004	−0.00085	+0.00078	0.976	0.7286
	7	−0.00001	−0.00116	+0.00114	0.976	0.9424
Total HOA RMS (µm)	3	−0.00092	−0.00222	+0.00038	0.991	<0.001 ***
	5	+0.00055	−0.00509	+0.00618	0.999	0.4584
	7	+0.00177	−0.01219	+0.01573	1.000	0.3358

Bias = mean difference (Cito−Prio); LoA = 95% limits of agreement; ICC = intraclass correlation coefficient. Statistical significance assessed by paired *t*-test, except for the following metrics where the Shapiro–Wilk test indicated non-normal distribution of differences and the Wilcoxon signed-rank test was used: RMS 3rd order (μm) at 3.00 mm; Sphere (D) at 5.00 mm; RMS 3rd order (μm) at 5.00 mm. * *p* < 0.05; *** *p* < 0.001.

**Table 5:** Two-way ANOVA: effect of front surface eccentricity and peripheral toricity on inter-device differences.

Metric	Aperture (mm)	FSE (*p*-Value)	TP (*p*-Value)	Interaction (*p*-Value)
Sphere (D)	3	0.387	0.553	0.138
	5	0.488	0.986	0.984
	7	0.202	0.830	0.796
RMS 3rd order (µm)	3	0.079	0.137	0.468
	5	0.159	0.984	0.521
	7	0.251	0.529	0.445
RMS 4th order (µm)	3	0.876	0.899	0.553
	5	0.915	0.583	0.804
	7	0.653	0.823	0.921
RMS 5th order (µm)	3	0.239	0.183	0.689
	5	0.693	0.344	0.270
	7	0.050	0.762	0.415
Total HOA RMS (µm)	3	0.079	0.191	0.722
	5	0.854	0.538	0.698
	7	0.662	0.823	0.962

Two-way ANOVA (Type II SS) on inter-device differences (Cito − Prio). FSE (front surface eccentricity): spherical designs (SS + ST, *n* = 8) vs. aspherical designs (AS + AT, FSE = 0.70, *n* = 8). TP (toric periphery): symmetric designs (SS + AS, *n* = 8) vs. toric peripheral. Interaction: tests whether the effect of FSE on inter-device differences depends on the presence of TP, and vice versa. Each factor uses all 16 lenses partitioned as SS (spherical symmetric), ST (spherical toric), AS (aspherical symmetric), AT (aspherical toric), *n* = 4 per group. *p* < 0.05.

## Data Availability

The data presented in this study are available on request from the corresponding author.
